# Cyclo­hexane-1,4-dicarb­oxy­lic acid–pyridinium-4-olate (1/1)

**DOI:** 10.1107/S160053681300754X

**Published:** 2013-03-28

**Authors:** Adriana Cruz-Enríquez, Hector J. Peinado-Guevara, Viviana Reyes-Marquez, Herbert Höpfl, José J. Campos-Gaxiola

**Affiliations:** a Facultad de Ingenieria Mochis, Universidad Autónoma de Sinaloa, Fuente Poseidón y Prol. A. Flores S/N, CP 81223, C.U. Los Mochis, Sinaloa, México; b Centro de Investigaciones Químicas, Universidad Autónoma del Estado de Morelos, Av. Universidad 1001, CP 62210, Cuernavaca, Morelos, México

## Abstract

In the title adduct, C_5_H_5_NO·C_8_H_12_O_4_, the heterocycle exists in its zwitterionic form. The cyclo­hexane ring exhibits a chair conformation with the carb­oxy­lic acid groups in equatorial and axial orientations. In the crystal, mol­ecules are linked through charge-assisted O—H⋯O^−^, N^+^—H⋯O^−^ and N^+^—H⋯O hydrogen bonds, and an additional series of C—H⋯O contacts, giving a pleated two-dimensional hydrogen-bonded network parallel to (-204).

## Related literature

For reports on supra­molecular crystal engineering and potential applications of co-crystals, see: Desiraju (1995 ▶); Simon & Bassoul (2000 ▶); Weyna *et al.* (2009 ▶); Aakeröy *et al.* (2010 ▶); Yan *et al.* (2012 ▶). For related structures, see: Bhogala *et al.* (2005 ▶); Shattock *et al.* (2008 ▶); Yu (2012 ▶).
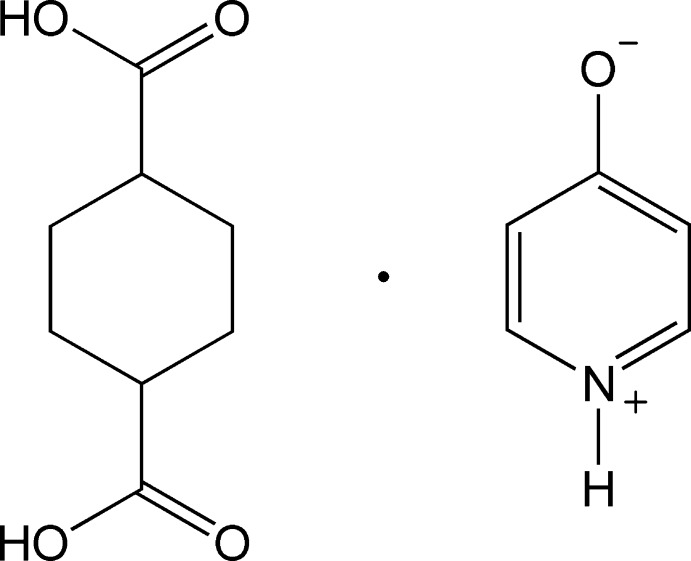


## Experimental

### 

#### Crystal data

C_5_H_5_NO·C_8_H_12_O_4_
*M* *_r_* = 267.28
Monoclinic, 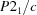

*a* = 11.749 (2) Å
*b* = 11.618 (2) Å
*c* = 10.8010 (19) Å
β = 115.383 (2)°
*V* = 1332.0 (4) Å^3^
*Z* = 4
Mo *K*α radiation
μ = 0.10 mm^−1^
*T* = 293 K
0.50 × 0.43 × 0.24 mm

#### Data collection

Bruker SMART CCD area-detector diffractometer
Absorption correction: multi-scan (*SADABS*; Sheldrick, 1996 ▶) *T*_min_ = 0.95, *T*_max_ = 0.98
12552 measured reflections
2345 independent reflections
2229 reflections with *I* > 2σ(*I*)
*R*_int_ = 0.041

#### Refinement

*R*[*F* ^2^ > 2σ(*F* ^2^)] = 0.072
*wR*(*F* ^2^) = 0.161
*S* = 1.02
2345 reflections
181 parameters
3 restraints
H atoms treated by a mixture of independent and constrained refinement
Δρ_max_ = 0.20 e Å^−3^
Δρ_min_ = −0.27 e Å^−3^

### 

Data collection: *SMART* (Bruker, 2000 ▶); cell refinement: *SAINT-Plus-NT* (Bruker, 2001 ▶); data reduction: *SAINT-Plus-NT*; program(s) used to solve structure: *SHELXTL-NT* (Sheldrick, 2008 ▶); program(s) used to refine structure: *SHELXTL-NT*; molecular graphics: *ORTEP-3* (Farrugia, 2012 ▶) and *Mercury* (Macrae *et al.* 2008 ▶); software used to prepare material for publication: *publCIF* (Westrip, 2010) ▶.

## Supplementary Material

Crystal structure: contains datablock(s) I, global. DOI: 10.1107/S160053681300754X/pk2472sup1.cif

Structure factors: contains datablock(s) I. DOI: 10.1107/S160053681300754X/pk2472Isup2.hkl

Supplementary material file. DOI: 10.1107/S160053681300754X/pk2472Isup3.cml

Additional supplementary materials:  crystallographic information; 3D view; checkCIF report

## Figures and Tables

**Table 1:** Hydrogen-bond geometry (Å, °)

*D*—H⋯*A*	*D*—H	H⋯*A*	*D*⋯*A*	*D*—H⋯*A*
O1—H1′⋯O5	0.84	1.82	2.638 (3)	165
O3—H3′⋯O5^i^	0.84	1.76	2.594 (2)	175
N1—H1⋯O4^ii^	0.84	2.29	2.921 (3)	132
N1—H1⋯O5^iii^	0.84	2.39	3.038 (3)	134
C1—H1*A*⋯O2^iv^	0.98	2.67	3.625 (4)	162
C11—H11⋯O2^iii^	0.93	2.62	3.420 (5)	143
C12—H12⋯O4^ii^	0.93	2.47	3.014 (4)	117

Symmetry codes: (i) 
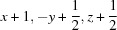
; (ii) 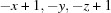; (iii) 
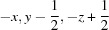
; (iv) 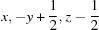.

**Fig. 1.**
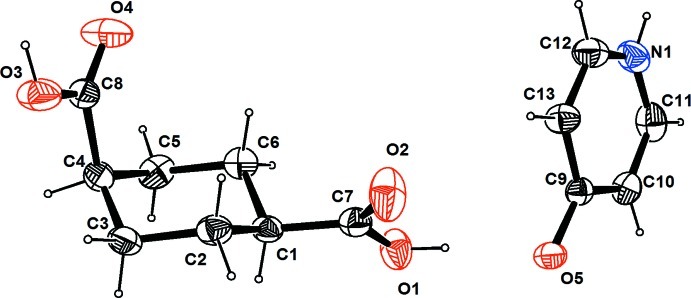
The molecular structures of the components in the title compound, showing displacement ellipsoids drawn at the 30% probability level.

**Fig. 2.**
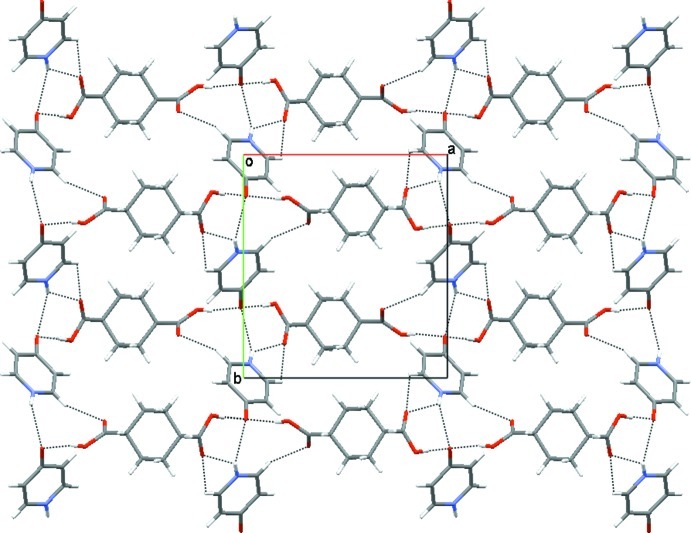
View down the c-axis of the two-dimensional hydrogen-bonded supramolecular network formed through O—H···O-, N+—H···O- and N+—H···O and C—H···O interactions.

## supplementary crystallographic information

### Comment

The engineering of novel materials *via* non-covalent synthesis has developed as a very attractive and potential area of research because of its importance in biological systems, molecular recognition (Simon *et al.*, 2000; Aakeröy *et al.*, 2010), pharmaceutical chemistry (Weyna *et al.*, 2009) and materials chemistry (Yan *et al.*, 2012). Aromatic carboxylic acids form reliable supramolecular synthons for the construction of novel organic networks by hydrogen bonding and π-π interactions (Desiraju, 1995), and numerous studies have focused on hydrogen bonding between carboxylic acids and pyridine derivatives (Bhogala *et al.*, 2005; Shattock *et al.* 2008; Yu, 2012). Herein, we report on the solid-state structure of a 1:1 co-crystal formed between cyclohexane-1,4-dicarboxylic acid and pyridin-4-ol. The molecular components of the title compound are shown in Fig. 1. The asymmetric unit contains one cyclohexane-1,4-dicarboxylic acid and one pyridin-4-ol molecule in the zwitterionic form. The cyclohexane ring exhibits a chair conformation with the carboxylic groups in equatorial and axial orientation, as denoted by the C7—C1—C2—C3 [-177.7 (2)°] and C8—C4—C5—C6 [75.7 (3)°] torsion angles, respectively. In the crystal, the molecular entities are linked through charge-assisted O—H···O^-^, N^+^—H···O^-^ and N^+^—H···O hydrogen bonds and an additional series of C—H···O contacts to give a pleated two-dimensional hydrogen-bonded network parallel to (-204) (Fig. 2, Table 1).

### Experimental

C_5_H_5_NO.C_8_H_12_O_4_ was prepared from a solution of C_5_H_5_NO (0.05 g, 0.53 mmol) and C_8_H_12_O_4_ (0.09 g, 0.53 mmol) in CH_3_OH (5 ml), which was stirred for a few minutes at room temperature, giving a clear transparent solution. After evaporation of the solvent, colorless crystals suitable for single-crystal X-ray diffraction had formed in about 51% yield. IR (KBr): 3471, 3276, 3131, 3092, 2937, 2863, 1709, 1632, 1576, 1509, 1416, 1364, 1331, 1316, 1229, 1170, 1001 cm^-1^.

### Refinement

H atoms were found in difference Fourier maps. Carbon-bound hydrogen atoms were placed in idealized positions using a riding models with constrained distances of 0.97 Å (R_2_CH_2_), 0.98 Å (R_3_CH) and 0.93 Å (C_sp2_H). Coordinates for hydrogens bound to oxygen and nitrogen were refined. U_iso_(H) values were set to either 1.2U_eq_ or 1.5U_eq_ (OH, NH) of the attached atom.

### Crystal data

**Table:**

C_5_H_5_NO·C_8_H_12_O_4_	*F*(000) = 568
*M**_r_* = 267.28	*D*_x_ = 1.333 Mg m^−^^3^
Monoclinic, *P*2_1_/*c*	Mo *K*α radiation, λ = 0.71073 Å
Hall symbol: -P 2ybc	Cell parameters from 5484 reflections
*a* = 11.749 (2) Å	θ = 2.6–27.4°
*b* = 11.618 (2) Å	µ = 0.10 mm^−^^1^
*c* = 10.8010 (19) Å	*T* = 293 K
β = 115.383 (2)°	Rectangular prism, yellow
*V* = 1332.0 (4) Å^3^	0.50 × 0.43 × 0.24 mm
*Z* = 4	

### Data collection

**Table:**

Bruker SMART CCD area-detector diffractometer	2345 independent reflections
Radiation source: fine-focus sealed tube	2229 reflections with *I* > 2σ(*I*)
Graphite monochromator	*R*_int_ = 0.041
φ and ω scans	θ_max_ = 25.0°, θ_min_ = 1.9°
Absorption correction: multi-scan (*SADABS*; Sheldrick, 1996)	*h* = −13→13
*T*_min_ = 0.95, *T*_max_ = 0.98	*k* = −13→13
12552 measured reflections	*l* = −12→12

### Refinement

**Table:**

Refinement on *F*^2^	Primary atom site location: structure-invariant direct methods
Least-squares matrix: full	Secondary atom site location: difference Fourier map
*R*[*F*^2^ > 2σ(*F*^2^)] = 0.072	Hydrogen site location: inferred from neighbouring sites
*wR*(*F*^2^) = 0.161	H atoms treated by a mixture of independent and constrained refinement
*S* = 1.02	*w* = 1/[σ^2^(*F*_o_^2^) + (0.0623*P*)^2^ + 1.4075*P*] where *P* = (*F*_o_^2^ + 2*F*_c_^2^)/3
2345 reflections	(Δ/σ)_max_ < 0.001
181 parameters	Δρ_max_ = 0.20 e Å^−^^3^
3 restraints	Δρ_min_ = −0.27 e Å^−^^3^

### Special details

**Table:**

Geometry. All e.s.d.'s (except the e.s.d. in the dihedral angle between two l.s. planes) are estimated using the full covariance matrix. The cell e.s.d.'s are taken into account individually in the estimation of e.s.d.'s in distances, angles and torsion angles; correlations between e.s.d.'s in cell parameters are only used when they are defined by crystal symmetry. An approximate (isotropic) treatment of cell e.s.d.'s is used for estimating e.s.d.'s involving l.s. planes.
Refinement. Refinement of *F*^2^ against ALL reflections. The weighted *R*-factor *wR* and goodness of fit *S* are based on *F*^2^, conventional *R*-factors *R* are based on *F*, with *F* set to zero for negative *F*^2^. The threshold expression of *F*^2^ > σ(*F*^2^) is used only for calculating *R*-factors(gt) *etc*. and is not relevant to the choice of reflections for refinement. *R*-factors based on *F*^2^ are statistically about twice as large as those based on *F*, and *R*- factors based on ALL data will be even larger.

### Fractional atomic coordinates and isotropic or equivalent isotropic displacement parameters (Å^2^)

**Table:**

	*x*	*y*	*z*	*U*_iso_*/*U*_eq_	
O1	0.2230 (2)	0.1930 (2)	0.0632 (2)	0.0663 (6)	
H1'	0.162 (2)	0.198 (4)	0.085 (4)	0.099*	
O2	0.3186 (2)	0.3059 (3)	0.2412 (3)	0.0898 (9)	
O3	0.8439 (2)	0.3327 (2)	0.3413 (2)	0.0733 (7)	
H3'	0.901 (3)	0.324 (4)	0.4210 (17)	0.110*	
O4	0.7984 (2)	0.15562 (19)	0.3701 (2)	0.0805 (8)	
C1	0.4258 (2)	0.2522 (2)	0.1037 (2)	0.0420 (6)	
H1A	0.3892	0.2539	0.0033	0.050*	
C2	0.5129 (3)	0.3552 (2)	0.1563 (3)	0.0489 (7)	
H2A	0.4647	0.4256	0.1246	0.059*	
H2B	0.5510	0.3555	0.2557	0.059*	
C3	0.6155 (3)	0.3511 (3)	0.1063 (3)	0.0556 (8)	
H3A	0.6718	0.4160	0.1442	0.067*	
H3B	0.5773	0.3583	0.0073	0.067*	
C4	0.6915 (3)	0.2400 (3)	0.1470 (3)	0.0497 (7)	
H4	0.7428	0.2374	0.0955	0.060*	
C5	0.6058 (3)	0.1347 (3)	0.1039 (3)	0.0557 (8)	
H5A	0.5683	0.1283	0.0048	0.067*	
H5B	0.6559	0.0662	0.1416	0.067*	
C6	0.5018 (3)	0.1405 (2)	0.1514 (3)	0.0485 (7)	
H6A	0.5384	0.1363	0.2506	0.058*	
H6B	0.4460	0.0751	0.1152	0.058*	
C7	0.3195 (3)	0.2542 (2)	0.1453 (3)	0.0471 (6)	
C8	0.7822 (2)	0.2371 (2)	0.2978 (3)	0.0442 (6)	
N1	0.0308 (3)	−0.0636 (2)	0.3628 (3)	0.0590 (7)	
H1	0.041 (4)	−0.115 (2)	0.421 (3)	0.088*	
O5	0.01175 (16)	0.18473 (15)	0.09311 (18)	0.0470 (5)	
C9	0.0146 (2)	0.1065 (2)	0.1791 (2)	0.0384 (6)	
C10	−0.0764 (3)	0.0194 (2)	0.1462 (3)	0.0495 (7)	
H10	−0.1444	0.0185	0.0603	0.059*	
C11	−0.0659 (3)	−0.0638 (2)	0.2390 (4)	0.0609 (8)	
H11	−0.1266	−0.1213	0.2159	0.073*	
C12	0.1179 (3)	0.0179 (3)	0.4004 (3)	0.0584 (8)	
H12	0.1836	0.0165	0.4880	0.070*	
C13	0.1124 (3)	0.1029 (2)	0.3127 (3)	0.0497 (7)	
H13	0.1740	0.1599	0.3410	0.060*	

### Atomic displacement parameters (Å^2^)

**Table:**

	*U*^11^	*U*^22^	*U*^33^	*U*^12^	*U*^13^	*U*^23^
O1	0.0561 (13)	0.0868 (16)	0.0593 (13)	−0.0227 (12)	0.0280 (11)	−0.0188 (12)
O2	0.0729 (16)	0.130 (2)	0.0779 (16)	−0.0249 (15)	0.0433 (14)	−0.0510 (16)
O3	0.0722 (15)	0.0658 (14)	0.0532 (13)	−0.0221 (12)	−0.0005 (11)	0.0129 (11)
O4	0.1024 (19)	0.0580 (14)	0.0471 (12)	−0.0139 (13)	−0.0003 (12)	0.0119 (11)
C1	0.0442 (14)	0.0476 (15)	0.0296 (12)	−0.0022 (11)	0.0115 (11)	0.0003 (11)
C2	0.0532 (16)	0.0370 (14)	0.0506 (15)	0.0012 (12)	0.0166 (13)	0.0067 (12)
C3	0.0529 (16)	0.0624 (18)	0.0448 (15)	−0.0094 (14)	0.0146 (13)	0.0164 (13)
C4	0.0479 (15)	0.0695 (19)	0.0349 (13)	0.0017 (14)	0.0207 (12)	0.0011 (13)
C5	0.0569 (17)	0.0591 (18)	0.0447 (15)	0.0045 (14)	0.0157 (13)	−0.0176 (13)
C6	0.0550 (16)	0.0374 (14)	0.0483 (15)	−0.0063 (12)	0.0175 (13)	−0.0069 (12)
C7	0.0480 (15)	0.0490 (16)	0.0400 (14)	−0.0005 (12)	0.0146 (12)	0.0019 (12)
C8	0.0437 (14)	0.0500 (16)	0.0386 (13)	0.0008 (12)	0.0174 (11)	0.0004 (12)
N1	0.0757 (18)	0.0480 (15)	0.0647 (17)	0.0093 (13)	0.0411 (15)	0.0138 (12)
O5	0.0441 (10)	0.0454 (10)	0.0432 (10)	−0.0014 (8)	0.0109 (8)	0.0082 (8)
C9	0.0411 (13)	0.0345 (13)	0.0403 (13)	0.0048 (10)	0.0182 (11)	−0.0015 (10)
C10	0.0450 (15)	0.0467 (16)	0.0554 (16)	−0.0027 (12)	0.0202 (13)	−0.0062 (13)
C11	0.0667 (19)	0.0407 (16)	0.088 (2)	−0.0111 (14)	0.0458 (19)	−0.0066 (15)
C12	0.0672 (19)	0.0558 (18)	0.0488 (16)	0.0103 (16)	0.0217 (14)	0.0081 (14)
C13	0.0517 (15)	0.0439 (15)	0.0449 (14)	−0.0043 (12)	0.0125 (12)	−0.0001 (12)

### Geometric parameters (Å, º)

**Table:**

O1—C7	1.310 (3)	C4—H4	0.9800
O1—H1'	0.8400 (10)	C5—C6	1.516 (4)
O2—C7	1.201 (3)	C5—H5A	0.9700
O3—C8	1.299 (3)	C5—H5B	0.9700
O3—H3'	0.8400 (11)	C6—H6A	0.9700
O4—C8	1.189 (3)	C6—H6B	0.9700
C1—C7	1.498 (4)	N1—C12	1.324 (4)
C1—C2	1.518 (4)	N1—C11	1.333 (4)
C1—C6	1.534 (4)	N1—H1	0.8400 (10)
C1—H1A	0.9800	O5—C9	1.289 (3)
C2—C3	1.518 (4)	C9—C10	1.403 (4)
C2—H2A	0.9700	C9—C13	1.408 (4)
C2—H2B	0.9700	C10—C11	1.359 (4)
C3—C4	1.524 (4)	C10—H10	0.9300
C3—H3A	0.9700	C11—H11	0.9300
C3—H3B	0.9700	C12—C13	1.351 (4)
C4—C8	1.517 (4)	C12—H12	0.9300
C4—C5	1.525 (4)	C13—H13	0.9300
			
C7—O1—H1'	112 (3)	H5A—C5—H5B	107.8
C8—O3—H3'	110 (3)	C5—C6—C1	111.2 (2)
C7—C1—C2	113.0 (2)	C5—C6—H6A	109.4
C7—C1—C6	110.6 (2)	C1—C6—H6A	109.4
C2—C1—C6	109.8 (2)	C5—C6—H6B	109.4
C7—C1—H1A	107.7	C1—C6—H6B	109.4
C2—C1—H1A	107.7	H6A—C6—H6B	108.0
C6—C1—H1A	107.7	O2—C7—O1	122.0 (3)
C3—C2—C1	110.7 (2)	O2—C7—C1	125.7 (3)
C3—C2—H2A	109.5	O1—C7—C1	112.3 (2)
C1—C2—H2A	109.5	O4—C8—O3	122.4 (2)
C3—C2—H2B	109.5	O4—C8—C4	124.3 (3)
C1—C2—H2B	109.5	O3—C8—C4	113.3 (2)
H2A—C2—H2B	108.1	C12—N1—C11	121.6 (3)
C2—C3—C4	112.4 (2)	C12—N1—H1	116 (3)
C2—C3—H3A	109.1	C11—N1—H1	122 (3)
C4—C3—H3A	109.1	O5—C9—C10	123.0 (2)
C2—C3—H3B	109.1	O5—C9—C13	121.1 (2)
C4—C3—H3B	109.1	C10—C9—C13	115.8 (2)
H3A—C3—H3B	107.9	C11—C10—C9	120.6 (3)
C8—C4—C3	112.5 (2)	C11—C10—H10	119.7
C8—C4—C5	112.2 (2)	C9—C10—H10	119.7
C3—C4—C5	111.2 (2)	N1—C11—C10	120.5 (3)
C8—C4—H4	106.8	N1—C11—H11	119.8
C3—C4—H4	106.8	C10—C11—H11	119.8
C5—C4—H4	106.8	N1—C12—C13	120.6 (3)
C6—C5—C4	112.6 (2)	N1—C12—H12	119.7
C6—C5—H5A	109.1	C13—C12—H12	119.7
C4—C5—H5A	109.1	C12—C13—C9	120.9 (3)
C6—C5—H5B	109.1	C12—C13—H13	119.6
C4—C5—H5B	109.1	C9—C13—H13	119.6
			
C7—C1—C2—C3	−177.7 (2)	C6—C1—C7—O1	−79.7 (3)
C6—C1—C2—C3	58.2 (3)	C3—C4—C8—O4	136.9 (3)
C1—C2—C3—C4	−56.6 (3)	C5—C4—C8—O4	10.5 (4)
C2—C3—C4—C8	−74.5 (3)	C3—C4—C8—O3	−44.6 (3)
C2—C3—C4—C5	52.4 (3)	C5—C4—C8—O3	−170.9 (2)
C8—C4—C5—C6	75.7 (3)	O5—C9—C10—C11	177.2 (2)
C3—C4—C5—C6	−51.4 (3)	C13—C9—C10—C11	−1.8 (4)
C4—C5—C6—C1	54.4 (3)	C12—N1—C11—C10	1.2 (4)
C7—C1—C6—C5	177.2 (2)	C9—C10—C11—N1	0.3 (4)
C2—C1—C6—C5	−57.4 (3)	C11—N1—C12—C13	−1.1 (5)
C2—C1—C7—O2	−22.5 (4)	N1—C12—C13—C9	−0.5 (4)
C6—C1—C7—O2	101.1 (3)	O5—C9—C13—C12	−177.1 (3)
C2—C1—C7—O1	156.7 (2)	C10—C9—C13—C12	1.9 (4)

### Hydrogen-bond geometry (Å, º)

**Table:**

*D*—H···*A*	*D*—H	H···*A*	*D*···*A*	*D*—H···*A*
O1—H1′···O5	0.84	1.82	2.638 (3)	165
O3—H3′···O5^i^	0.84	1.76	2.594 (2)	175
N1—H1···O4^ii^	0.84	2.29	2.921 (3)	132
N1—H1···O5^iii^	0.84	2.39	3.038 (3)	134
C1—H1*A*···O2^iv^	0.98	2.67	3.625 (4)	162
C11—H11···O2^iii^	0.93	2.62	3.420 (5)	143
C12—H12···O4^ii^	0.93	2.47	3.014 (4)	117

Symmetry codes: (i) *x*+1, −*y*+1/2, *z*+1/2; (ii) −*x*+1, −*y*, −*z*+1; (iii) −*x*, *y*−1/2, −*z*+1/2; (iv) *x*, −*y*+1/2, *z*−1/2.
